# Structural transition in Bcl-xL and its potential association with mitochondrial calcium ion transport

**DOI:** 10.1038/srep10609

**Published:** 2015-05-29

**Authors:** Sreekanth Rajan, Minjoo Choi, Quoc Toan Nguyen, Hong Ye, Wei Liu, Hui Ting Toh, CongBao Kang, Neelagandan Kamariah, Chi Li, Huiya Huang, Carl White, Kwanghee Baek, Gerhard Grüber, Ho Sup Yoon

**Affiliations:** 1 School of Biological Sciences, Nanyang Technological University, 60 Nanyang Drive, Singapore 637551; 2 Experimental Therapeutics Centre, Agency for Science, Technology and Research (A*STAR), Singapore 138669; 3 Bioinformatics Institute, Agency for Science, Technology and Research (A*STAR), 30 Biopolis Street, Singapore 138671; 4 Molecular Targets Program, James Graham Brown Center and Department of Medicine, Pharmacology and Toxicology, University of Louisville, Louisville, KY 40202, USA; 5 Department of Physiology and Biophysics, Rosalind Franklin University of Medicine and Science, 3333 Green Bay Road, North Chicago, IL 60064, USA; 6 Department of Genetic Engineering, College of Life Sciences, Kyung Hee University, Yongin-si, Gyeonggi-do, 446-701, Republic of Korea

## Abstract

Bcl-2 family proteins are key regulators for cellular homeostasis in response to apoptotic stimuli. Bcl-xL, an antiapoptotic Bcl-2 family member, undergoes conformational transitions, which leads to two conformational states: the cytoplasmic and membrane-bound. Here we present the crystal and small-angle X-ray scattering (SAXS) structures of Bcl-xL treated with the mild detergent *n*-Octyl β-D-Maltoside (OM). The detergent-treated Bcl-xL forms a dimer through three-dimensional domain swapping (3DDS) by swapping helices α6-α8 between two monomers. Unlike Bax, a proapoptotic member of the Bcl-2 family, Bcl-xL is not converted to 3DDS homodimer upon binding BH3 peptides and ABT-737, a BH3 mimetic drug. We also designed Bcl-xL mutants which cannot dimerize and show that these mutants reduced mitochondrial calcium uptake in MEF cells. This illustrates the structural plasticity in Bcl-xL providing hints toward the probable molecular mechanism for Bcl-xL to play a regulatory role in mitochondrial calcium ion transport.

The Bcl-2 family of proteins is a key apoptotic regulator in response to a wide variety of stimuli perturbing cellular homeostasis^1^,^2^. Apoptosis is a tightly regulated process through dynamic molecular interactions among multiple networks of proteins including the Bcl-2 family. The antiapoptotic Bcl-2 proteins interact with the proapoptotic Bcl-2 family members and prevent the formation of an oligomeric pore in the mitochondrial outer membrane (MOM)^3^. The proapoptotic Bax exists latent and monomeric in the cytosol before the induction of apoptosis^4^,^5^. Bax/Bak molecule itself or complex formation with other ligands requires membrane binding and conformational rearrangements to induce apoptosis^6^,^7^,^8^,^9^. Notable structural transitions and homo-oligomerization of Bax have been observed in the presence of detergents for MOM permeabilization (MOMP)^10^,^11^. In contrast, Bcl-xL primarily resides in the mitochondrial outer membrane with the C-terminal helix embedded at the mitochondrial membrane and induces small, transient alterations of the membrane permeability without affecting consequences for the MOMP^12^. Despite the difference in the pore formation, Bcl-xL still can interfere Bax’s membrane insertion and MOMP steps^4^,^12^, suggesting that Bax and Bcl-xL may harness distinct molecular mechanisms in inducing structural rearrangements with respect to their relevant biological functions. Apart from modulating MOMP directly, the Bcl-2 family proteins and Bcl-xL in particular regulate the calcium homeostasis through interactions with Voltage Gated Anion Channel (VDAC), a MOM protein^13^,^14^. The molecular determinants in Bcl-xL which are necessary for regulating calcium homeostasis at the MOM are still obscure. On the other hand, recent crystal structures of Bax and Bak revealed how detergents and BH3 domains induce Bax / Bak homodimerization and consequently trigger them to an activated oligomeric form to induce apoptosis^11^,^15^. Despite evidence that cellular Bcl-xL exists in two topological states^16^, structural studies have been mainly restricted to monomers, either alone or in complex with the BH3 domain peptides derived from the Bcl-2 family^17^,^18^,^19^,^20^. To delineate the poorly understood structural transition steps between water-soluble and membrane-bound conformations of Bcl-xL and ultimately the molecular mechanism of Bcl-xL, we determined the X-ray crystal structure of Bcl-xL in the presence of the detergent *n*-Octyl β-D-Maltoside (OM). The overall topology of the detergent-induced Bcl-xL is a 3D domain-swapped (3DDS) homodimer comparable with that of Bcl-xL induced by high pH^21^ and Bax induced by the same detergent^11^. The BH3 peptides inhibit this OM induced Bcl-xL dimer formation, as assessed by NMR spectroscopy and Blue Native PAGE (BN-PAGE). This is in contrast with the proapoptotic Bax where the BH3 peptides induced 3DDS homodimer formation in the presence of detergents^11^. In addition, we show that the BH3 mimetic drug, ABT-737 also inhibits Bcl-xL dimer formation. Our biophysical studies reveal how Bcl-xL undergoes a monomer-dimer transition through a three-dimensional domain swapping (3DDS) mechanism and suggest a molecular model for the inhibition of this structural rearrangement which we have correlated toward its probable role in calcium homeostasis.

## Results

### Dimerization of Bcl-xL

Oligomerization of Bcl-2 family of proteins is an essential step toward mitochondrial outer membrane insertion^21^,^22^. Previously it was shown that Bcl-xL (ΔCT) when mixed with synthetic liposomes could be recovered as 3DDS dimer^21^. The full-length Bcl-xL containing the long flexible loop (L) and the hydrophobic transmembrane C-terminal domain (CT) exhibits limited solubility and heavily overlapping NMR resonances (Supplementary Information Figures S1a and S1b). As the full-length Bcl-xL (Bcl-xLFL) and the C-terminal and loop-truncated Bcl-xL (Bcl-xL∆L∆CT) show similar structural folds and BH3 peptide binding profiles, as assessed by two-dimensional (2D) ^1^H-^15^N TROSY-HSQC and HSQC spectra (Supplementary Information Figure S1), we used the soluble Bcl-xL∆L∆CT (referred to as Bcl-xL hereafter) for our structural studies. *n*-Octyl β-D-Maltoside (OM) was the detergent used to study the oligomerization properties of Bcl-xL. The formation of a dimer induced by the addition of 2% OM was confirmed by crosslinking (Supplementary Information Figure S2) using 1, 6-Bis(maleimido)hexane (BMH) which crosslinks the cysteine (C151) residue present in the structure whose distance is unique in 3DDS dimer conformation. Further using crystallography, NMR, Small angle X-ray scattering (SAXS) and BN-PAGE we have characterized the dimerization of Bcl-xL.

### X-ray structure reveals that Bcl-xL rearranges its conformation to a domain-swapped dimer

**Figure 1:**
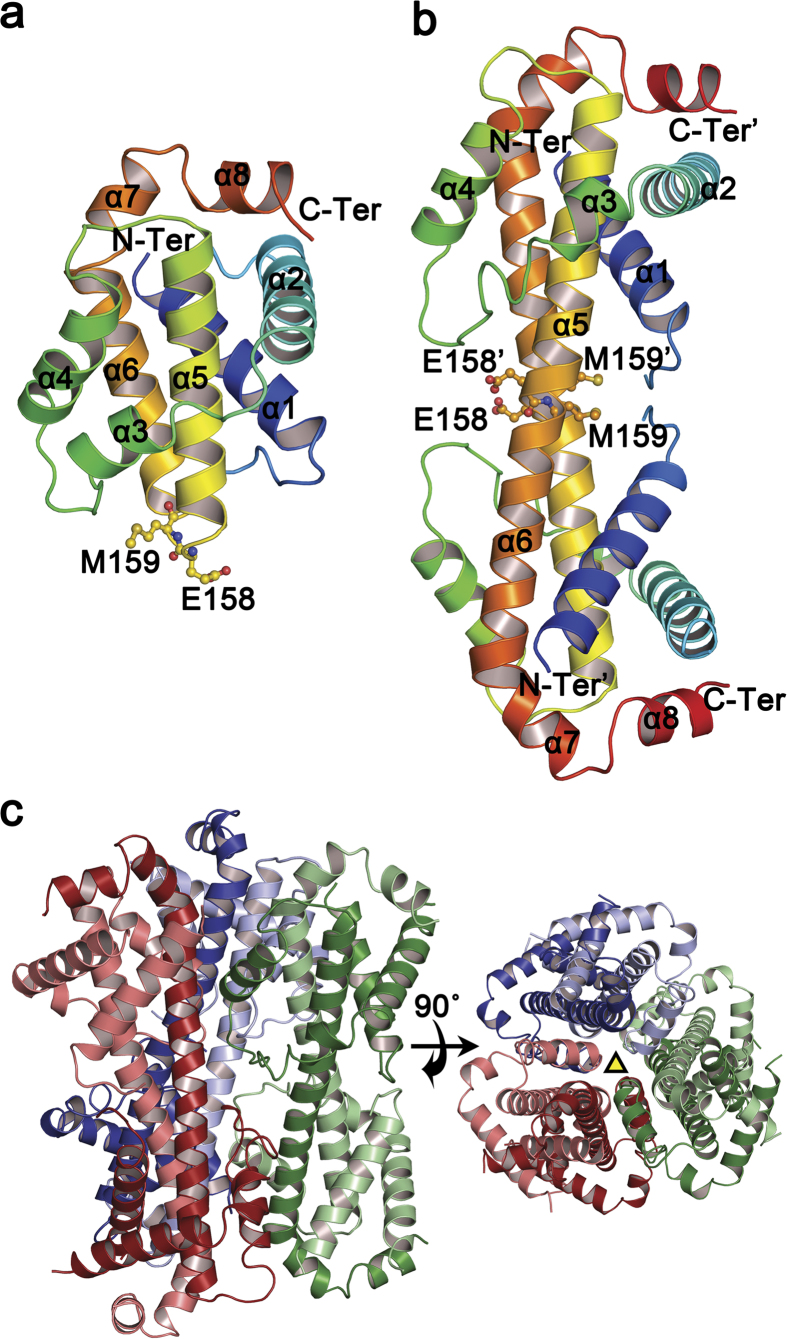
The crystal structure of Bcl-xL induced by the detergent OM. (**a**) The cartoon representation of the monomer unit is shown (for reference), colored in accordance with their position (N-terminal in blue to C-terminal in red), and their α-helices are labeled. The residues Glu158 and Met159 are shown in ball and stick mode. (**b**) The 3D Domain Swapped (3DDS) dimer can be seen clearly as the N- and C-terminal regions of the two chains swap against each other with helices α5 and α6 fusing into a single helix. (**c**) The cartoon representation of the hexamer, a trimer of dimers, observed from crystal packing. The three dimers are colored red/pale red, green/pale green and blue/pale blue. A rotation by 90 ° is shown indicating the location of the three fold crystallographic rotation axis (yellow colored triangle).

**Figure 2:**
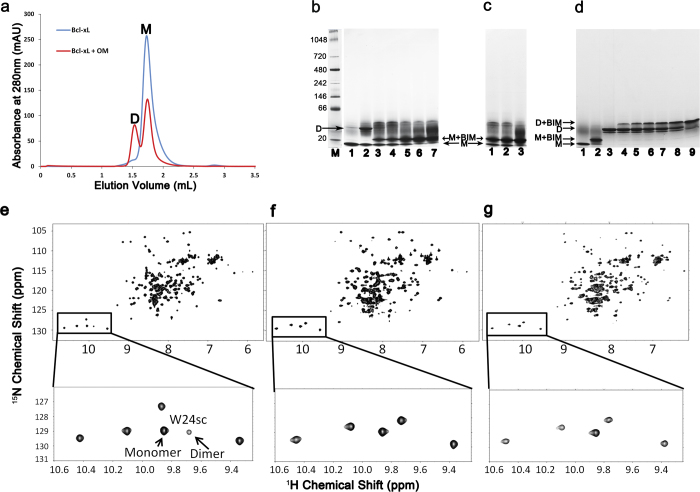
Oligomerization and BH3 binding. (**a**) The OM-treated Bcl-xL (red line) was eluted by size exclusion chromatography using Superdex 75 15/150 GL, while that without OM was run as control (blue line). The dimer (D) and monomer (M) peaks are indicated. (**b**) The BN-PAGE showing the Bcl-xL monomer (lane 1) and dimer (lane 2) form. The addition of 2% OM and BIM to Bcl-xL, incubated overnight, at increasing concentrations of BIM (1:0.5 (lane 3), 1:1 (lane 4), 1:2.5 (lane 5), 1:5 (lane 6) and 1:10 (lane 7)), reveals that BIM inhibits OM from inducing the Bcl-xL dimers, while the band corresponding to the monomer-BIM complex could be observed above the monomer band. (**c)** It could be seen that incubation of BIM to Bcl-xL at molar ratios of 1:2.5 (lane 1), 1:5 (lane 2) and 1:10 (lane 3) followed by the addition of OM, also inhibits the dimer formation. From ‘b’ and ‘c’ it could be concluded that BH3 peptide binding to monomeric Bcl-xL arrests its conversion to the dimeric form. (**d**) Unlike in ‘b’ and ‘c’, purified Bcl-xL dimer was incubated with increasing concentrations of BIM (1:0.2 (lane 4), 1:0.4 (lane 5), 1:0.6 (lane 6), 1:0.8 (lane 7), 1:1 (lane 8) and 1:2 (lane 9)). The presence of dimer-BIM complex is visible by a small shift of the equivalent dimer band. The presence of BIM in the Bcl-xL-BIM complex bands was confirmed by protein ID analysis using mass spectroscopy. (**e**) 2D ^1^H-^15^N HSQC spectrum of the 0.2 mM ^15^N-labeled Bcl-xL in the presence of 2% OM. Sidechain amide proton of tryptophan’s is highlighted in a zoomed view of the region. Peaks representing both monomeric and dimeric W24 are marked. (**f**) 2D ^1^H-^15^N HSQC spectrum showing ^15^N-labeled Bcl-xL with 2% CHAPS and a 20-fold excess of BIM peptide. No additional sidechain amide proton of tryptophan residues, other than the ones from the monomeric form of the Bcl-xL, was detected. (**g**) 2D ^1^H-^15^N HSQC spectrum showing the ^15^N-labeled Bcl-xL with 5-fold excess of BIM followed by addition of 2% OM, where the dimer was not observed.

To gain insights into how Bcl-xL undergoes structural rearrangements in the presence of a detergent, we crystallized Bcl-xL treated with OM. The overall fold of Bcl-xL induced by the detergent OM reveals a domain-swapped dimer (Figs. 1a,b) conformation, which is topologically identical to that reported for 3DDS Bax dimers induced by OM^11^ and Bcl-xL exposed to an alkaline pH (pH 10)^21^. In the dimeric conformation helices α6-α8 of one Bcl-xL molecule are reciprocally exchanged with α6′-α8′ of the other (Fig. 1b), retaining the hydrophobic binding pocket exposed to solvent. The helices α5 and α6 in the water-soluble structure^20^ fuse into a single long helix, which interacts with the equivalent helix from the neighboring unit oriented in an anti-parallel direction. The dimer interface (monomer-monomer) area, calculated by the software PISA^23^, was ~3215 Å^2^ made up of a total of 79 residues. This indicates that the dimer unit forms a stable building block, as observed from the gel filtration (Fig. 2a) and supported by small angle X-ray scattering (SAXS) data analysis (see below).

In the structure presented here, the crystal symmetry generated a hexameric assembly, a trimer of dimers (Fig. 1c), which was not observed in the previously reported pH induced dimer^21^. But the dimer-dimer interface is weak indicating that the hexamer could be a transient oligomer or just an effect of crystal packing.

### Low resolution solution structure of 3DDS Bcl-xL by small angle X-ray scattering (SAXS)

**Figure 3:**
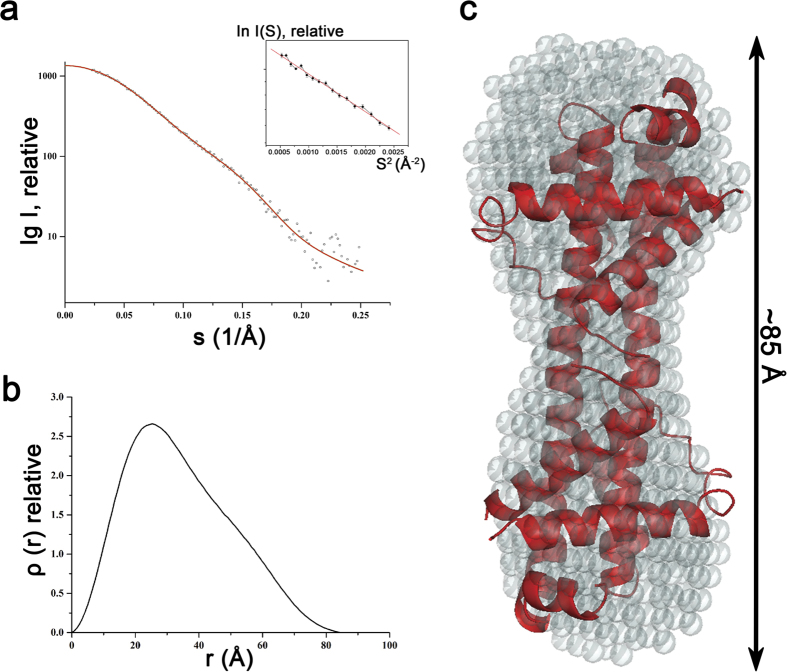
Small Angle X-ray Scattering (SAXS) of Bcl-xL dimer induced by OM. (**a**) Small angle X-ray scattering pattern (ο) and its corresponding fitting curves (green - experimental, red - calculated). The Guinier plot from this scattering curve (shown as an inset) reveals that the protein is not aggregated. (**b**) The pair distribution function (ρ(r)) plot indicating a D_max_ of ~85 Å. (**c**) The *ab initio* shape of the solution structure the Bcl-xL dimer (grey spheres) with a length of ~85 Å, is shown to fit well with the crystal structure of the Bcl-xL 3DDS dimer (red colored cartoon representation).

To verify proper three dimensional folding and to determine the first low resolution solution structure of Bcl-xL treated with OM, SAXS patterns of purified Bcl-xL dimers, induced by OM, were recorded. The final composite scattering curve indicates a monodispersed protein in solution (Fig. 3a). Inspection of the low angle of the Guinier plots reveals a good data quality and no protein aggregation (*inset* of Fig. 3a). Bcl-xL has a radius of gyration (*R*_*g*_) of 26.66 ± 0.2 Å and a maximum dimension (*D*_*max*_) of 84.39 ± 0.2 Å (Fig. 3b). Comparison of the forward scattering of Bcl-xL with the values obtained from a reference solution of Lysozyme (14.39 kDa) yields a molecular mass of 41.51 kDa, indicating that Bcl-xL is dimeric at the concentrations used. The solution *R*_*g*_ and *D*_*max*_ values match well with the dimeric crystal structure *R*_*g*_ value of 24.5 Å and the *D*_*max*_ of 79.6 Å.

The SAXS solution structure of Bcl-xL was restored *ab initio* from the scattering patterns. The obtained shape for the protein yielded a good fit to the experimental data in the entire scattering range (Fig. 3c). The corresponding fit has a discrepancy of χ^2^ = 0.958. The Bcl-xL shape is elongated with a total length of 85.3 Å and slide globular domains at both ends (Fig. 3c). Bcl-xL solution shape determined by SAXS superimposed well with the crystallographic 3DDS dimer structure of Bcl-xL with an r.m.s. deviation of 0.93 Å (Fig. 3c). The SAXS solution structure has not only enabled us to cross validate the dimer conformation observed in the crystal structure but also helped us in showing that the Bcl-xL dimer adopts a stable solution conformation.

### Comparison of Bcl-xL with Bax structures

**Figure 4:**
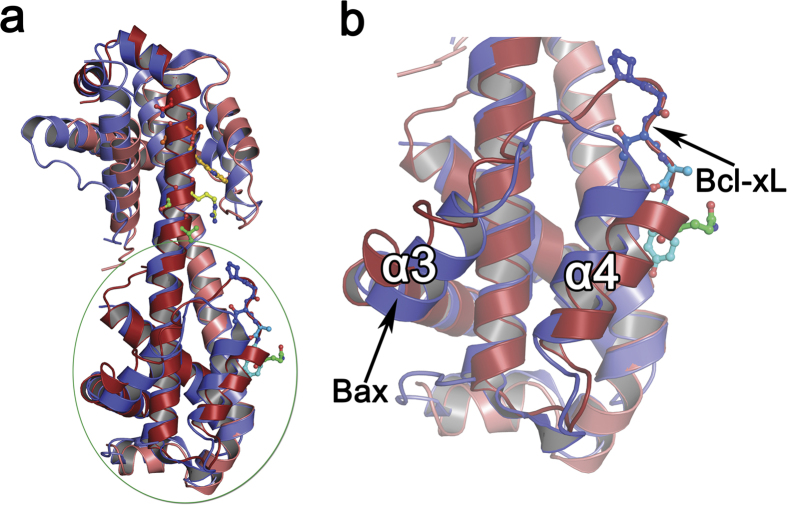
Comparison of Bcl-xL and Bax 3DDS structures. (**a**) Superposition of Bcl-xL (red, pale red) on Bax (blue, pale blue) reveals visible structural changes in the α3-α4 loop and α4 helix orientation. (**b**) A closer view projects these changes, with α4 (red) of Bcl-xL moving away from the BH3 pocket by ~15 ° compared to that of the Bax structure (blue). The α3 which is in general a distorted region in the Bcl-2 family also shows some changes.

Structural superposition of the Bcl-xL 3DDS dimer with Bax 3DDS dimer (Fig. 4a) reveals that the BH3 binding pocket has been altered probably as a reflection of the extended α3-α4 loop (Fig. 4b) in Bcl-xL. The helix α4 of Bcl-xL moves away from the pocket by ~15 ° compared to the Bax structure, owing to the α3-α4 loop extension. A structure based sequence alignment^24^,^25^ with Bax reconfirmed that the α3-α4 loop is elongated in Bcl-xL (Supplementary Information Figure S3). Similarly a major conformational change is observed in the α5-α6 loop, which converts to an alpha helix in the dimeric form. This is due to a change in the backbone torsion angle’s phi and psi of the residue E158 (Supplementary Information Table S1) which was earlier reported in the pH-induced 3DDS dimer as well^21^. Interestingly, the corresponding residue in the Bax structure (Supplementary Information Figure S3) is the positively charged K128 which also shows a similar switch of the torsion angles (Supplementary Information Table S1), indicating that this loop and in particular the residue E158 / K128 could serve as the threshold position to overcome the energetic barrier nucleating such a large conformational change in these structures.

### BH3 peptides inhibit Bcl-xL dimerization

**Figure 5:**
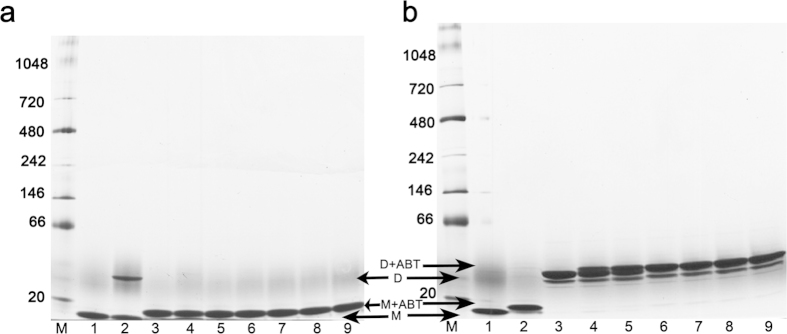
ABT-737 inhibits Bcl-xL dimer formation. (**a**) BN-PAGE showing that pre-incubated ABT-737 with Bcl-xL prevents the detergent OM from inducing dimerization. Lanes 1, 2 and 3 are the apo Bcl-xL, OM induced dimer and apo with ABT-737 (1:5 molar ratio) as controls respectively. Then ABT-737 was incubated with Bcl-xL in increasing molar ratio’s of 1:0.5 (lane 4), 1:1 (lane 5), 1:1.5 (lane 6), 1:2 (lane 7), 1:2.5 (lane 8), 1:5 (lane 9) for 2 hrs followed by the addition of 2% OM. This reveals that ABT-737 arrests the monomer from converting to a dimer. (**b**) In a similar way, purified dimer (lane 3) induced by OM was incubated with increasing concentrations of ABT-737 (1:0.2 (lane4), 1:0.4 (lane 5), 1:0.6 (lane 6), 1:0.8 (lane 7), 1:1.0 (lane 8), 1:2 (lane 9)). Lanes 1, 2 and 3 shows the Bcl-xL monomer, monomer with ABT-737 (1:2 molar ratio) and purified dimer as controls respectively. The protein markers are labeled as lane M in both ‘a’ and ‘b’.

Bax induced a structural transition from monomer to dimer in response to the detergent CHAPS together with BH3 peptide^11^. To understand whether the structural rearrangements featuring Bcl-xL’s dimer formation have functional implication in apoptotic regulation, we examined the role of BIM BH3 peptide in this inter-conversion by employing BN-PAGE and NMR spectroscopy on a 2D ^1^H-^15^N HSQC spectrum of the ^15^N-labeled, OM induced dimeric Bcl-xL. Previously it has been reported that the BID BH3 peptide slows down the inter-conversion of monomer to dimer induced by heat^26^ and that the Bcl-xL dimer formation can be easily identified by the observation of an upfield movement of the sidechain amide proton of W24 (W24sc) in a 1D ^1^H NMR spectrum^26^. By using W24sc as a probe, dimeric Bcl-xL was detected in 2D ^1^H-^15^N HSQC spectrum of the ^15^N-labeled Bcl-xL with the addition of 2% OM, though monomeric Bcl-xL is predominant as indicated by the peak intensity of the monomeric and dimeric W24sc in this condition (Fig. 2e). Adding more OM into the mixture had no effect on increasing the percentage of the Bcl-xL dimer (data not shown). Our NMR (Fig. 2f) and BN-PAGE (Supplementary Information Figure S4) results showed that Bcl-xL treated with CHAPS plus BH3 peptides does not induce dimer formation, unlike Bax. These results suggest that Bcl-xL and Bax may employ distinct molecular mechanisms in inducing structural rearrangements in membranes. It is evident that the proapoptotic BH3 peptides, in the present case BIM, prevent the conversion of monomer to dimer (Figs. 2b,c). However, the BIM BH3 peptide can bind with pre-formed dimer (Fig. 2d), implying that the hydrophobic pocket is accessible in the dimer conformation, which is also observed in the crystal structure. It is well known that in the membrane, Bcl-xL is known to inhibit Bax oligomerization^6^,^27^,^28^, while the BH3 peptides enhance Bax oligomerization^11^. Interestingly, the BH3-mimetic drug ABT-737 also inhibits the conversion of Bcl-xL monomer to dimer, similar to the BIM BH3 peptide. BN-PAGE (Fig. 5) shows that Bcl-xL incubated with ABT-737 clearly arrests the monomer from converting to a dimer, whereas there are no monomers observed when ABT-737 was added to the purified dimer, indicating that the drug behaves like the BIM BH3 mimetic (Figs. 2b–d) toward the Bcl-xL dimer as well.

### Mutations in the α5-α6 loop region affect physiological function of Bcl-xL

**Figure 6:**
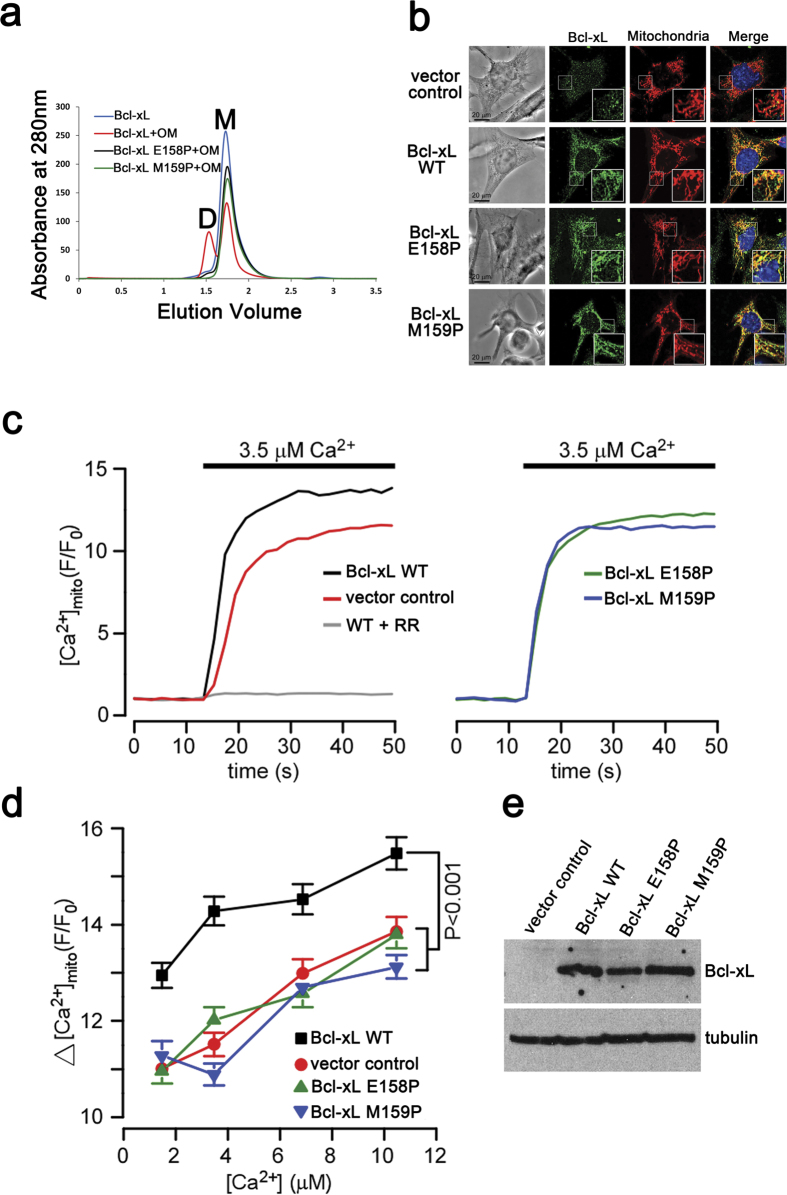
Residues E158 and M159 are essential for Bcl-xL to regulate mitochondrial ion homeostasis. (**a**) Size exclusion chromatography column Superdex 75 15/150 GL (3 ml) profile revealing that the mutations E158P (black line) and M159P (green line) do not induce Bcl-xL dimerization. The untreated (blue line) and OM treated (red line) Bcl-xL are shown as controls to indicate the monomer (‘M’) and dimer (‘D’) peak positions. (**b**) The mitochondrial localization images after expressing the empty vector, WT or Bcl-xL mutants (E158P & M159P) in Bcl-xL knockout MEF cells confirm that the mutants also localize in the mitochondria similar to WT Bcl-xL. The scale bar is 20 μm. (**c**) Representative traces showing [Ca^2+^]_mito_ in permeabilized Bcl-xL-KO cells expressing empty vector, WT or Bcl-xL mutants (E158P & M159P) during step increases in external [Ca^2+^] from zero to 3.5 μM in the presence or absence of ruthenium red (10 μM; RR). (**d**) Summary of ∆[Ca^2+^]_mito_ in response to physiological changes (1.5, 3.5, 6.9 and 10 μM) in external [Ca^2+^]. Data were pooled from six independent imaging experiments carried out on three different days (mean ± SEM; two-way ANOVA). (**e**) Western blot confirming the expression of wild type, E158P and M159P Bcl-xL in the Bcl-xL-KO background. Representative of two independent trials are shown.

Given 3DDS dimer-mediated structural rearrangement of Bcl-xL involves the α5-α6 loop region, we examined how mutations in the α5-α6 loop could affect the molecular characteristics and functioning of Bcl-xL. We chose the residues E158 and M159 in this region (Fig. 1a), the former undergoes a drastic torsion angle rotation (Supplementary Information Table S1) and the latter being its neighboring residue. These two residues were mutated to Proline, a well-known helix breaker. The mutations E158P and M159P arrested the OM induced dimer formation of Bcl-xL (Fig. 6a), indicating that the α5-α6 loop region is critical for Bcl-xL dimer formation. In addition, to validate that these mutations did not affect Bcl-xL folding, we carried out NMR experiments, which not only confirmed that they remain folded but also show that they can bind the BIM BH3 peptide (Supplementary Information Figure S5), reaffirming that the Bcl-xL mutants retain a monomer-like fold along with the conserved binding pocket.

Then we assessed the involvement of Bcl-xL and its mutant forms in regulating the physiological ion transport in mitochondria (Figs. 6b–e). Bcl-xL knockout MEF cells^29^ were stably transfected with Bcl-xL wild type, Bcl-xL mutants (E158P, M159P) or empty vector (Fig. 6e). The mitochondrial localization of E158P and M159P was confirmed using immunocytochemistry (Fig. 6b). Mitochondrial Ca^2+^([Ca^2+^]_mito_) uptake was monitored in these cell lines using the Ca^2+^ indicator rhod-2 in digitonin-permeabilized cells, as described previously^14^. After equilibration in Ca^2+^-free buffer containing mitochondrial substrates, [Ca^2+^]_mito_ uptake was evoked by exposing cells to physiologically relevant Ca^2+^ concentrations (Figs. 6c,d). Sensitivity to the [Ca^2+^]_mito_ uptake blocker ruthenium red (RR) confirmed that the observed fluorescent changes were exclusively mitochondrial in origin. Compared to vector control, the expression of wild type Bcl-xL on the knockout background was associated with a markedly larger [Ca^2+^]_mito_ uptake in response to the entire range of Ca^2+^ concentrations (Fig. 6d), consistent with previous observations^14^. In contrast, expression of the Bcl-xL mutants (E158P or M159P) on the knockout background had no effect on [Ca^2+^]_mito_ uptake. Correlating this with our *in vitro* biophysical studies, we presume that the conformational changes associated with dimerization may be essential to Bcl-xL’s role in mitochondrial Ca^2+^ homeostasis. On hindsight, cell based experiments are required to validate the physiological existence of Bcl-xL dimerization at MOM.

## Discussion

Different types of three-dimensional domain swapping have been reported in a diverse range of proteins^30^,^31^,^32^. Interestingly, the 3DDS dimerization of Bax mediated by detergents and BH3 peptides and Bcl-xL in an alkaline condition or elevated temperature were recently discovered^11^,^21^,^26^. It was proposed^21^ that the 3DDS dimerization could be the probable conformational switch necessary for the membrane insertion of Bcl-xL, though debated. Therefore we attempted to determine the structure of Bcl-xL using the detergent OM to mimic membrane environment. The OM induced dimer resulted in a notable monomer-dimer transition, whereas non-physiological conditions such as alkaline pH or heat (Supplementary Information Figure S6 ) induced little transitions, suggesting that the detergent-induced dimer might be a more stable form of Bcl-xL and mimics a near-physiological condition. The structure of the OM-induced dimer of Bcl-xL determined using X-ray crystallography and validated by SAXS is presented here. The SAXS data confirms that the dimer is not a crystallographic artifact. Earlier, the structural similarity of Bcl-xL with the pore forming toxins, the diphtheria toxin and colicin, led to the hypothesis that Bcl-xL could form pores in the mitochondrial outer membrane^18^,^33^,^34^,^35^,^36^. Whether the hexamer, observed due to crystal packing (Fig. 1c), could be a possible physiological higher oligomeric state adopted by Bcl-xL needs to be characterized further. We also show that BIM BH3 peptide inhibits Bcl-xL dimerization, based on BN-PAGE and NMR studies, possibly to protect Bax oligomerization^37^ in the membrane by (a) engaging the Bcl-xL dimers, thereby preventing Bcl-xL from inhibiting Bax oligomerization or (b) a step ahead where they inhibit the Bcl-xL conversion from monomers to dimers.

Nonetheless, we speculate that structural transitions associated with dimerization may serve as a primary step toward membrane insertion which could propagate to other higher oligomers^21^,^26^,^38^,^39^. We show that the mutations E158P and M159P (Fig. 6a) in the α5-α6 loop of Bcl-xL arrest this dimer formation. These mutants also show reduced Ca^2+^([Ca^2+^]_mito_ ) uptake (Figs. 6b–d) in cells, indicating that the Bcl-xL which cannot dimerize could reduce calcium ion regulation in the MOM. The structural plasticity of Bcl-xL underlying this transition may suggest a Bcl-xL-mediated mitochondrial Ca^2+^ homeostasis.

Various models have been proposed toward the orientation and insertion mechanism of the Bcl-2 family proteins in the MOM^11^,^40^, but remain fully unresolved to-date. Anyhow, oligomerization of Bcl-2 proteins remains a vital necessity towards this step. Though the 3DDS conformation of Bax has been shown to be off-pathway^11^, the significance of this conformation in Bcl-xL needs further validation. Although we cannot claim that Bcl-xL adopts a 3DDS dimer conformation in MOM, structural changes related to dimerization could be a possible conformational transition necessary for this, as with other Bcl-2 family members. In a similar situation, a BH3-in-groove model was proposed for membrane insertion of proapoptotic Bax^22^ and Bak^15^,^22^,^41^,^42^ which may be improbable for Bcl-xL’s opposing function and also the affinity of BH3 motif of Bcl-xL on its own hydrophobic pocket is feeble^20^. On the contrary, it was recently shown that the BH3 motif of Bcl-xL can bind its own pocket, where they do not rule out a BH3-in-groove model for Bcl-xL^43^. It is to be noted that the other antiapoptotic protein Mcl-1 can also binds its own BH3 domain^44^. To this end, we require more studies on homodimerization of Mcl-1 and other antiapoptotic Bcl-2 proteins to expose the versatile biological functions within the members of the Bcl-2 protein family. Presently, it is ambiguous to arrive at a consensus on the oligomerization, orientation and insertion mechanism of Bcl-xL in the MOM, requiring additional studies inclusive of other antiapoptotic Bcl-2 family proteins as well.

In conclusion, we have characterized the detergent-induced oligomerization of Bcl-xL using biophysical methods and correlated this with their probable role in MOM calcium homeostasis. It is known that detergents can induce Bcl-xL dimerization, but we have determined their structure using X-ray crystallography and SAXS. BH3 peptides, ABT-737 and the mutations E158P / M159P have been shown to be factors arresting Bcl-xL dimerization. In addition, we show that the mutant Bcl-xL which cannot dimerize, observed from our biophysical studies, reduced Ca^2+^([Ca^2+^]_mito_ ) uptake in MEF cells. The molecular basis of the Bcl-xL interactome underlying this mechanism needs further investigation. From our data, we surmise that Bcl-xL homodimerization or conformational changes associated with it could be a plausible transition toward its function in regulating calcium homoeostasis at the MOM. Anyhow, the physiological existence of Bcl-xL dimerization / oligomerization at MOM needs to be addressed to ascertain our premise.

### Experimental Procedures

#### Cloning, expression and purification

Bcl-xL construct used in this study lacks the C-terminal transmembrane region and a flexible loop as previously described^20^. The corresponding cDNA fragment was cloned into the pETSUMO vector (LifeSensors, USA). The plasmid containing the gene was transformed into *E. coli* BL21 (DE3) cells. The cells were then grown at 37 ˚C in LB media with 30 μg/ml kanamycin until absorbance at 600 nm reached 0.6, after which the protein expression was induced at 25 °C for 4 hours by adding Isopropyl β-D-1-thiogalactopyranoside (IPTG) to a final concentration of 1 mM. The cells were then harvested by centrifugation at 8,000 *g* for 15 min. The pellets were suspended in resuspension buffer (50 mM Tris-HCl pH 7.8, 150 mM NaCl, 5 mM 2-mercaptoethanol, 2 mM PMSF) and lysed by sonication for 30 min on ice. The cell lysate was cleared by ultracentrifugation at 20,000 *g* for 30 minutes. The supernatant containing the protein was then purified using Ni^2+^-NTA column chromatography using a linear imidazole gradient of 0–500 mM. Small Ubiquitin-like Modifier (SUMO) tag was cleaved by the addition of SUMO protease at 18 °C overnight. The SUMO tag-cleaved sample was loaded on to a second Ni^2+^-NTA affinity column to get rid of the SUMO tag and SUMO protease followed by gel filtration using a HiLoad 16/60 Superdex 200 column (GE Healthcare, Sweden). The purified Bcl-xL was incubated with 2% OM overnight to induce dimer formation. A two-step chromatography purification using an ion exchange Resource Q 1 ml column (GE Healthcare) followed by gel filtration HiLoad 16/60 Superdex 75 column was carried out to obtain homogenous Bcl-xL dimer species which were used for BN-PAGE and SAXS experiments. For NMR studies, isotopically-labeled Bcl-xL proteins were expressed in *E. coli* BL21 (DE3) cells that were grown in an M9 medium containing ^15^NH_4_Cl and purified by Ni^2+^-affinity followed by a HiLoad 16/60 Superdex 200 column chromatography. For the preparation of uniformly ^15^N, ^2^H-labeled full-length Bcl-xL, cells were grown in D_2_O. Samples were in 20 mM sodium phosphate, pH 7.0, 50 mM NaCl, 0.8 mM TCEP and 5% ^2^H_2_O.

#### Crosslinking of Bcl-xL after dimerization

Bcl-xL was concentrated to 10 mg/ml and incubated with 2% OM to induce dimers. Induced Bcl-xL dimers were then crosslinked at cysteine residues (C151) by incubation with 1 mM 1, 6-Bis(maleimido)hexane (BMH, Pierce) for 1 h at room temperature. Crosslinking reaction was quenched by 4 mM DTT. Later, the presence of the dimer was confirmed using 15% SDS-PAGE.

#### Blue Native PAGE

Oligomerization of Bcl-xL in the presence of detergents, BIM BH3 peptide and ABT-737 were analyzed using the Novex Bis-Tris gel system (Invitrogen) according to the manufacture’s protocol.

#### Small Angle X-ray Scattering (SAXS)

Small angle X-ray scattering (SAXS) data of Bcl-xL were measured at the NANOSTAR instrument (Bruker), equipped with a Metal-jet X-ray source and Vantec 2000-detector system. The metal-jet source use the liquid gallium source to deliver a high intensity X-ray beam at the wavelength of λ = 1.34 Å. The SAXS-measurement were carried out with the source to sample distance of 145 cm, a two pinhole collimation system and the sample to detector distance of 67 cm. SAXS experiments have been carried out with 1 mg/ml and 3 mg/ml of Bcl-xL in a sample volume of 40 μl at 15 °C. For each measurement, a total of six measurements at 5 min interval were recorded. The data were flood-field, spatial corrected and processed using the in-build SAXS software. We tested the possible radiation damage by comparing the six data sets and no changes were detected. The scattering of the buffer was subtracted and the difference curves were scaled for the concentration. All the data processing steps were performed automatically using the program package PRIMUS^45^. The forward scattering *I(0)* and the radius of gyration *R*_*g*_ were evaluated using the Guinier approximation^46^. These parameters were also computed from the entire scattering patterns using the indirect transform package GNOM^47^, which also provide the distance distribution function *p(r).* Low-resolution models of the protein were built by the program DAMMIN^48^. *Ab initio* solution shapes of Bcl-xL were obtained by superposition of ten independent model reconstructions with the program package SUBCOMP^49^ and building an averaged model from the most probable one using the DAMAVER program^50^.

#### Crystallization and Structure determination

Purified Bcl-xL at 10 mg/ml concentration incubated with 2% OM overnight was subjected to crystal screening. Diffraction quality crystals appeared with 1.0 M sodium citrate and 0.1 M CHES pH 9.0 in the reservoir and equal volumes of protein and reservoir in a 4 μl drop, using hanging drop vapour diffusion method. The crystals diffracted to 2.8 Å resolution at 100 K on beamline 13B1 at the National Synchrotron Radiation Research Center (Hsinchu, Taiwan) using an ADSC-Quantum 315 detector, using 20% glycerol as the cryo protectant added to the mother liquor. The data was indexed, integrated, merged and scaled using the software *i*Mosflm^51^ and SCALA^52^. The crystals belonged to the cubic space group I 4_1_ 3 2, with one molecule in the asymmetric unit, which was identified using the program PHASER^53^ with the molecular replacement model PDB ID 2B48^21^. The programs PHENIX^54^ and COOT^55^ were used for refinement and map fitting respectively. The final R-factor and R-free converged to 0.236 and 0.268 respectively. One glycerol molecule was observed near the C-terminal of helix α6. The program MOLPROBITY^56^ showed 98.5% of the residues are in allowed regions of the Ramachandran plot^57^. The data collection and refinement statistics are given in Supplementary Information Table S2.

#### NMR spectroscopy

All NMR experiments were performed at 310K on a Bruker AVANCE II 700 MHz NMR spectrometer equipped with four RF channels and a 5 mm z-gradient TXI cryoprobe. The chemical shifts were referenced to internal 2,2-dimethyl-2-silapentane-5-sulfonate acid (DSS). 2D ^1^H-^15^N TROSY-HSQC and HSQC spectra were acquired using standard Bruker pulse sequences with 8 or 32 scans, 2,048 × 256 complex points and spectral windows of 16 ppm x 40 ppm in the ^1^H and ^15^N dimensions, respectively. All NMR spectra were processed by NMRPipe^58^ and displayed using Sparky (Goddard, T.D. & Kneller, D.G. SPARKY 3. *University of California, San Francisco*).

#### Cell lines and culture

The generation of mouse embryonic fibroblast (MEF) cells from wild-type (WT) and Bcl-xL knockout (KO) embryos, as well as selection of cell lines stably expressing WT and mutant Bcl-xL in the KO background, have been described previously^29^. MEF cells were maintained in Dulbecco’s Modified Eagle Medium (DMEM) medium (Mediatech; Manassas, VA) supplemented with 10% Fetal Bovine Serum (FBS) (Gemini; West Sacramento, CA), 100 U ml^−1^ penicillin (Mediatech), and 100 μg ml^−1^ streptomycin (Mediatech) in an incubator with 95% humidity and 5% CO_2_ at 37 ^°^C.

#### Mitochondrial [Ca^2+^] measurement

Wide-field fluorescent microscopy was used to measure [Ca^2+^]_mito_ in plasma membrane-permeabilized preparations. Cells cultured on glass coverslips were loaded with 3 μM Rhod-2 AM (Life Technologies; Grand Island, NY) by incubation at 37 ^o^C for 30 minutes and mounted in a recording chamber on the stage of an inverted IX71 microscope (Olympus America Inc., Center Valley, PA). Cells were excited at 548 nm and emitted fluorescence was filtered at 605 nm and collected using a CCD-based imaging system running SimplePCI software (Hamamatsu Corporation; Sewickley, PA). The chamber was continuously perfused with physiological buffer at room temperature, and a rapid solution changer used to switch the composition of the solution bathing the cells under study. Cells were permeabilized by 3-4 minutes exposure to digitonin (Sigma-Aldrich; St. Louis, MO; 25 μg ml^−1^) applied in Ca^2+^-free intracellular like media (ICM) with the composition (mM): KCl (120), NaCl (10), KH_2_PO_4_ (1), HEPES (20), sodium succinate (2), EGTA (1), pH 7.1 with KOH. The permeabilized preparation was then allowed to equilibrate in Ca^2+^-free ICM for 15 minutes prior to recording. Experimental solutions containing varying free Ca^2+^ concentrations were made by adjusting the ratio of Ca^2+^/HEDTA, calculated using maxchelator (C. Patton, Stanford University, CA).

#### Data collection and analysis

Data were collected using a 20x objective enabling capture of ~100 cells per image field. To compare differences between cell lines, cells were cultured at the same density and passaged in parallel, and data were acquired on the same day. Fluorescence intensity changes were background-subtracted and normalized to the initial fluorescence value F_0_ and expressed as F/F_0_. Data were summarized as mean ± SEM. A two-way ANOVA with Fisher’s LSD (Least Significant Difference) post hoc analysis was employed for multiple comparisons and differences between means were accepted as statistically significant at the 95% level (P < 0.05).

#### Immunocytochemistry

Bcl-xL knockout MEF cells stably expressing Bcl-xL WT, Bcl-xL mutants (E158P, M159P) or empty vector were cultured on glass coverslips. Cells were fixed with 4% paraformaldehyde and permeabilized with 0.3% Triton X-100. Bcl-xL was labeled with monoclonal antibody (1:1000; BD Biosciences; San Jose, CA) and detected by secondary labeling with green-fluorescent Alexa Fluor 488 IgG (1:750 dilution; Invitrogen). Mitochondria were visualized in the same cells using CellLight® Mitochondria-RFP (Invitrogen) according to the manufacturer’s instructions. Coverslips were mounted and counterstained with DAPI using ProLong® Gold Antifade Mountant (Invitrogen). Images were acquired using an Olympus FV10i confocal microscope and processed with Fluoview1000 software (Olympus America Inc.).

#### Accession Numbers

PDB accession number for the crystal structure reported in this study is 4PPI.

## Additional Information

**How to cite this article**: Rajan, S. *et al.* Structural transition in Bcl-xL and its potential association with mitochondrial calcium ion transport. *Sci. Rep.* **5**, 10609; doi: 10.1038/srep10609 (2015).

## Supplementary Material

Supporting Information

## References

[b1] Youle R. J. & Strasser A. The BCL-2 protein family: opposing activities that mediate cell death. Nat. Rev. Mol. Cell Biol. 9, 47–59 (2008). 18097445 10.1038/nrm2308

[b2] Rathmell J. C. & Thompson C. B. Pathways of apoptosis in lymphocyte development, homeostasis, and disease. Cell 109 Suppl, S97–107 (2002). 11983156 10.1016/s0092-8674(02)00704-3

[b3] Oltvai Z. N., Milliman C. L. & Korsmeyer S. J. Bcl-2 heterodimerizes in vivo with a conserved homolog, Bax, that accelerates programmed cell death. Cell 74, 609–619 (1993). 8358790 10.1016/0092-8674(93)90509-o

[b4] Hsu Y. T., Wolter K. G. & Youle R. J. Cytosol-to-membrane redistribution of Bax and Bcl-X(L) during apoptosis. Proc. Natl. Acad. Sci. U. S. A. 94, 3668–3672 (1997). 9108035 10.1073/pnas.94.8.3668 PMC20498

[b5] Wolter K. G. *et al.* Movement of Bax from the cytosol to mitochondria during apoptosis. J. Cell Biol. 139, 1281–1292 (1997). 9382873 10.1083/jcb.139.5.1281 PMC2140220

[b6] Dlugosz P. J. *et al.* Bcl-2 changes conformation to inhibit Bax oligomerization. EMBO J. 25, 2287–2296 (2006). 16642033 10.1038/sj.emboj.7601126 PMC1478188

[b7] Kim P. K., Annis M. G., Dlugosz P. J., Leber B. & Andrews D. W. During apoptosis bcl-2 changes membrane topology at both the endoplasmic reticulum and mitochondria. Mol. Cell 14, 523–529 (2004). 15149601 10.1016/s1097-2765(04)00263-1

[b8] Gavathiotis E., Reyna D. E., Davis M. L., Bird G. H. & Walensky L. D. BH3-triggered structural reorganization drives the activation of proapoptotic BAX. Mol. Cell 40, 481–492 (2010). 21070973 10.1016/j.molcel.2010.10.019 PMC3050027

[b9] Moldoveanu T. *et al.* BID-induced structural changes in BAK promote apoptosis. Nat. Struct. Mol. Biol. 20, 589–597 (2013). 23604079 10.1038/nsmb.2563 PMC3683554

[b10] Suzuki M., Youle R. J. & Tjandra N. Structure of Bax: coregulation of dimer formation and intracellular localization. Cell 103, 645–654 (2000). 11106734 10.1016/s0092-8674(00)00167-7

[b11] Czabotar P. E. *et al.* Bax crystal structures reveal how BH3 domains activate Bax and nucleate its oligomerization to induce apoptosis. Cell 152, 519–531 (2013). 23374347 10.1016/j.cell.2012.12.031

[b12] Bleicken S., Wagner C. & Garcia-Saez A. J. Mechanistic differences in the membrane activity of Bax and Bcl-xL correlate with their opposing roles in apoptosis. Biophys. J. 104, 421–431 (2013). 23442864 10.1016/j.bpj.2012.12.010 PMC3552256

[b13] Arbel N., Ben-Hail D. & Shoshan-Barmatz V. Mediation of the antiapoptotic activity of Bcl-xL protein upon interaction with VDAC1 protein. J. Biol. Chem. 287, 23152–23161 (2012). 22589539 10.1074/jbc.M112.345918 PMC3391160

[b14] Huang H. *et al.* An interaction between Bcl-xL and the voltage-dependent anion channel (VDAC) promotes mitochondrial Ca2+ uptake. J. Biol. Chem. 288, 19870–19881 (2013). 23720737 10.1074/jbc.M112.448290 PMC3707689

[b15] Brouwer J. M. *et al.* Bak Core and Latch Domains Separate during Activation, and Freed Core Domains Form Symmetric Homodimers. Mol. Cell 55, 938–946 (2014). 25175025 10.1016/j.molcel.2014.07.016

[b16] Jeong S. Y. *et al.* Bcl-x(L) sequesters its C-terminal membrane anchor in soluble, cytosolic homodimers. EMBO J. 23, 2146–2155 (2004). 15131699 10.1038/sj.emboj.7600225 PMC424420

[b17] Liu X., Dai S., Zhu Y., Marrack P. & Kappler J. W. The structure of a Bcl-xL/Bim fragment complex: implications for Bim function. Immunity 19, 341–352 (2003). 14499110 10.1016/s1074-7613(03)00234-6

[b18] Muchmore S. W. *et al.* X-ray and NMR structure of human Bcl-xL, an inhibitor of programmed cell death. Nature 381, 335–341 (1996). 8692274 10.1038/381335a0

[b19] Petros A. M. *et al.* Rationale for Bcl-xL/Bad peptide complex formation from structure, mutagenesis, and biophysical studies. Prot. Sci. 9, 2528–2534 (2000). 10.1110/ps.9.12.2528 PMC2144516 11206074

[b20] Sattler M. *et al.* Structure of Bcl-xL-Bak peptide complex: recognition between regulators of apoptosis. Science 275, 983–986 (1997). 9020082 10.1126/science.275.5302.983

[b21] O’Neill J. W., Manion M. K., Maguire B. & Hockenbery D. M. BCL-XL dimerization by three-dimensional domain swapping. J. Mol. Biol. 356, 367–381 (2006). 16368107 10.1016/j.jmb.2005.11.032

[b22] Czabotar P. E., Lessene G., Strasser A. & Adams J. M. Control of apoptosis by the BCL-2 protein family: implications for physiology and therapy. Nat. Rev. Mol. Cell Biol. 15, 49–63 (2014). 24355989 10.1038/nrm3722

[b23] Krissinel E. & Henrick K. Inference of macromolecular assemblies from crystalline state. J. Mol. Biol. 372, 774–797 (2007). 17681537 10.1016/j.jmb.2007.05.022

[b24] Pettersen E. F. *et al.* UCSF Chimera--a visualization system for exploratory research and analysis. J. Comput. Chem. 25, 1605–1612 (2004). 15264254 10.1002/jcc.20084

[b25] Gouet P., Robert X. & Courcelle E. ESPript/ENDscript: Extracting and rendering sequence and 3D information from atomic structures of proteins. Nucl. Acids Res. 31, 3320–3323 (2003). 12824317 10.1093/nar/gkg556 PMC168963

[b26] Denisov A. Y., Sprules T., Fraser J., Kozlov G. & Gehring K. Heat-induced dimerization of BCL-xL through alpha-helix swapping. Biochemistry (Mosc). 46, 734–740 (2007). 10.1021/bi062080a 17223694

[b27] Billen L. P., Kokoski C. L., Lovell J. F., Leber B. & Andrews D. W. Bcl-XL inhibits membrane permeabilization by competing with Bax. PLoS Biol. 6, e147 (2008). 18547146 10.1371/journal.pbio.0060147 PMC2422857

[b28] Parikh N. *et al.* The N-terminus and alpha-5, alpha-6 helices of the pro-apoptotic protein Bax, modulate functional interactions with the anti-apoptotic protein Bcl-xL. BMC Cell Biol. 8, 16 (2007). 17519046 10.1186/1471-2121-8-16 PMC1890283

[b29] Eno C. O. *et al.* Distinct roles of mitochondria- and ER-localized Bcl-xL in apoptosis resistance and Ca2+ homeostasis. Mol. Biol. Cell 23, 2605–2618 (2012). 22573883 10.1091/mbc.E12-02-0090 PMC3386223

[b30] Liu Y. & Eisenberg D. 3D domain swapping: as domains continue to swap. Prot. Sci. 11, 1285–1299 (2002). 10.1110/ps.0201402 PMC2373619 12021428

[b31] Liu Y., Gotte G., Libonati M. & Eisenberg D. A domain-swapped RNase A dimer with implications for amyloid formation. Nat. Struct. Biol. 8, 211–214 (2001). 11224563 10.1038/84941

[b32] Liu Y., Hart P. J., Schlunegger M. P. & Eisenberg D. The crystal structure of a 3D domain-swapped dimer of RNase A at a 2.1-A resolution. Proc. Natl. Acad. Sci. U. S. A. 95, 3437–3442 (1998). 9520384 10.1073/pnas.95.7.3437 PMC19854

[b33] Schendel S. L. *et al.* Channel formation by antiapoptotic protein Bcl-2. Proc. Natl. Acad. Sci. U. S. A. 94, 5113–5118 (1997). 9144199 10.1073/pnas.94.10.5113 PMC24640

[b34] Minn A. J. *et al.* Bcl-x(L) forms an ion channel in synthetic lipid membranes. Nature 385, 353–357 (1997). 9002522 10.1038/385353a0

[b35] Schlesinger P. H. *et al.* Comparison of the ion channel characteristics of proapoptotic BAX and antiapoptotic BCL-2. Proc. Natl. Acad. Sci. U. S. A. 94, 11357–11362 (1997). 9326614 10.1073/pnas.94.21.11357 PMC23466

[b36] Schendel S. L., Montal M. & Reed J. C. Bcl-2 family proteins as ion-channels. Cell Death Differ. 5, 372–380 (1998). 10200486 10.1038/sj.cdd.4400365

[b37] Wang X., Xing D., Liu L. & Chen W. R. BimL directly neutralizes Bcl-xL to promote Bax activation during UV-induced apoptosis. FEBS Lett. 583, 1873–1879 (2009). 19427863 10.1016/j.febslet.2009.04.045

[b38] Westphal D., Kluck R. M. & Dewson G. Building blocks of the apoptotic pore: how Bax and Bak are activated and oligomerize during apoptosis. Cell Death Differ. 21, 196–205 (2014). 24162660 10.1038/cdd.2013.139 PMC3890949

[b39] Pagliari L. J. *et al.* The multidomain proapoptotic molecules Bax and Bak are directly activated by heat. Proc. Natl. Acad. Sci. U. S. A. 102, 17975–17980 (2005). 16330765 10.1073/pnas.0506712102 PMC1312392

[b40] Losonczi J. A. *et al.* NMR studies of the anti-apoptotic protein Bcl-xL in micelles. Biochemistry (Mosc). 39, 11024–11033 (2000). 10.1021/bi000919v 10998239

[b41] Aluvila S. *et al.* Organization of the Mitochondrial Apoptotic BAK Pore: Oligomerization of the BAK homodimers. J. Biol. Chem. 289, 2537–2551 (2014). 24337568 10.1074/jbc.M113.526806 PMC3908389

[b42] Ma S. *et al.* Assembly of the Bak apoptotic pore: a critical role for the Bak protein alpha6 helix in the multimerization of homodimers during apoptosis. J. Biol. Chem. 288, 26027–26038 (2013). 23893415 10.1074/jbc.M113.490094 PMC3764807

[b43] Lee E. F. *et al.* The Functional Differences between Pro-survival and Pro-apoptotic B Cell Lymphoma 2 (Bcl-2) Proteins Depend on Structural Differences in Their Bcl-2 Homology 3 (BH3) Domains. J. Biol. Chem. 289, 36001–36017 (2014). 25371206 10.1074/jbc.M114.610758 PMC4276867

[b44] Stewart M. L., Fire E., Keating A. E. & Walensky L. D. The MCL-1 BH3 helix is an exclusive MCL-1 inhibitor and apoptosis sensitizer. Nat. Chem. Biol. 6, 595–601 (2010). 20562877 10.1038/nchembio.391 PMC3033224

[b45] Svergun D. I. A Direct Indirect Method of Small-Angle Scattering Data Treatment. J. Appl. Crystallogr. 26, 258–267 (1993).

[b46] Guinier A. & Fournet G. Small-angle Scattering of X-rays. Wiley, New York (1955).

[b47] Svergun D. I. Determination of the Regularization Parameter in Indirect-Transform Methods Using Perceptual Criteria. J. Appl. Crystallogr. 25, 495–503 (1992).

[b48] Svergun D. I. Restoring low resolution structure of biological macromolecules from solution scattering using simulated annealing. Biophys J 76, 2879–2886 (1999). 10354416 10.1016/S0006-3495(99)77443-6 PMC1300260

[b49] Kozin M. B. & Svergun D. I. Automated matching of high- and low-resolution structural models. J. Appl. Crystallogr. 34, 33–41 (2001).

[b50] Volkov V. V. & Svergun D. I. Uniqueness of ab initio shape determination in small-angle scattering. J. Appl. Crystallogr. 36, 860–864 (2003). 10.1107/S0021889809000338 PMC5023043 27630371

[b51] Battye T. G., Kontogiannis L., Johnson O., Powell H. R. & Leslie A. G. iMOSFLM: a new graphical interface for diffraction-image processing with MOSFLM. Acta Crystallogr. D. Biol. Crystallogr. 67, 271–281 (2011). 21460445 10.1107/S0907444910048675 PMC3069742

[b52] Evans P. Scaling and assessment of data quality. Acta Crystallogr. D. Biol. Crystallogr. 62, 72–82 (2006). 16369096 10.1107/S0907444905036693

[b53] McCoy A. J. *et al.* Phaser crystallographic software. J. Appl. Crystallogr. 40, 658–674 (2007). 19461840 10.1107/S0021889807021206 PMC2483472

[b54] Adams P. D. *et al.* PHENIX: a comprehensive Python-based system for macromolecular structure solution. Acta Crystallogr. D. Biol. Crystallogr. 66, 213–221 (2010). 20124702 10.1107/S0907444909052925 PMC2815670

[b55] Emsley P. & Cowtan K. Coot: model-building tools for molecular graphics. Acta Crystallogr. D. Biol. Crystallogr. 60, 2126–2132 (2004). 15572765 10.1107/S0907444904019158

[b56] Chen V. B. *et al.* MolProbity: all-atom structure validation for macromolecular crystallography. Acta Crystallogr. D. Biol. Crystallogr. 66, 12–21 (2010). 20057044 10.1107/S0907444909042073 PMC2803126

[b57] Ramachandran G. N., Ramakrishnan C. & Sasisekharan V. Stereochemistry of polypeptide chain configurations. J. Mol. Biol. 7, 95–99 (1963). 13990617 10.1016/s0022-2836(63)80023-6

[b58] Delaglio F. *et al.* NMRPipe: a multidimensional spectral processing system based on UNIX pipes. J. Biomol. NMR 6, 277–293 (1995). 8520220 10.1007/BF00197809

