# Supplementary material for: Structural transition in Bcl-xL and its potential association with mitochondrial calcium ion transport
Source: Sci Rep. 2015 May 29;5:10609. doi: 10.1038/srep10609 (PMC4448555; doi:10.1038/srep10609)
Supplement: Supporting Information [file srep10609-s1.pdf]

Supplementary Information

Structural transition in Bcl-xL and its potential association with mitochondrial calcium ion transport

Sreekanth Rajan[1], Minjoo Choi[1], Quoc Toan Nguyen[1], Hong Ye[1], Wei Liu[1], Hui Ting Toh[1], CongBao Kang[2], Neelagandan Kamariah[3], Gerhard Grüber[1,3], Chi Li[4], Huiya Huang[5], Carl White[5], Kwanghee Baek[6], and Ho Sup Yoon[1,6¶]

[1]School of Biological Science, Nanyang Technological University, 60 Nanyang Drive, Singapore 637665

[2]Experimental Therapeutics Centre, Agency for Science, Technology and Research, Singapore 138669

[3]Bioinformatics Institute, Agency for Science, Technology and Research (A*STAR), 30 Biopolis Street, Singapore 138671

[4]Molecular Targets Program, James Graham Brown Center and Department of Medicine, Pharmacology and Toxicology, University of Louisville, Louisville, KY 40202, USA

[5]Department of Physiology and Biophysics, Rosalind Franklin University of Medicine and Science, 3333 Green Bay Road, North Chicago, IL 60064, USA

[6]Department of Genetic Engineering, College of Life Sciences, Kyung Hee University Yongin-si, Gyeonggi-do, 446-701, Republic of Korea

Author Contributions: H.S.Y conceived the study. S.R., M.C., N.Q.T., H.Y., W.L., H.T.T., C.K., N.K., and H.H. performed the experiments. S.R., M.C., H.Y., C.K., G.G., C.L., C.W., K.B., and H.S.Y. analyzed the data. S.R., G.G., C.L., C.W., and H.S.Y. wrote the manuscript.

[¶]To whom correspondence should be addressed: hsyoon@ntu.edu.sg ; Telephone: +65-6316 2846 ; Fax: + 65 6791-3856

Supplementary Figures

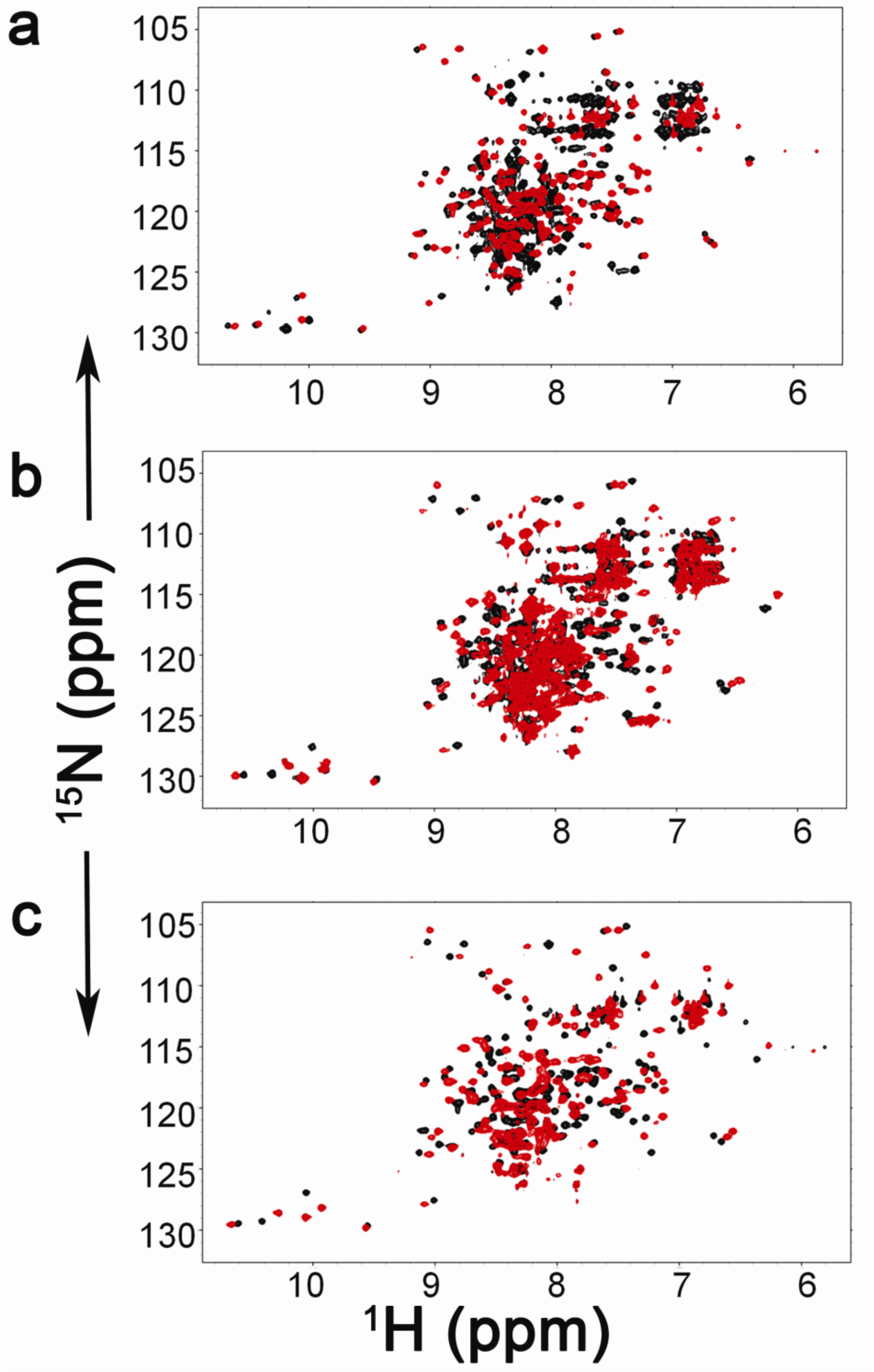

Figure S1: NMR comparison of the full-length Bcl-xL (Bcl-xLFL), the C-terminal and loop-truncated Bcl-xL (Bcl-xL) and their BH3 peptide binding profiles in the presence of CHAPS at 310K. (a) Overlay of 2D ^{1}H-^{15}N TROSY- HSQC spectrum of 0.2 mM ^{15}N, ^{2}H-labeled Bcl-xLFL purified with CHAPS (black) and 2D ^{1}H-^{15}N HSQC spectrum of 0.2 mM ^{15}N-labeled

Bcl-xL saturated with 2 % CHAPS (red). (b) Overlay of 2D ^{1}H-^{15}N TROSY- HSQC spectra of 0.2 mM ^{15}N, ^{2}H-labeled Bcl-xLFL with CHAPS in the absence (black) and presence (red) of BIM BH3 peptide at a 6:1 molar ratio of BIM BH3 to the Bcl-xLFL, where Bcl-xL was saturated with BIM BH3. (c) Overlay of 2D ^{1}H-^{15}N HSQC spectra of 0.2 mM ^{15}N-labeled Bcl-xL with 2 % CHAPS in the absence (black) and presence (red) of BIM BH3 peptide at a 4:1 molar ratio of BIM BH3 to Bcl-xL, where Bcl-xL was saturated with BIM BH3.

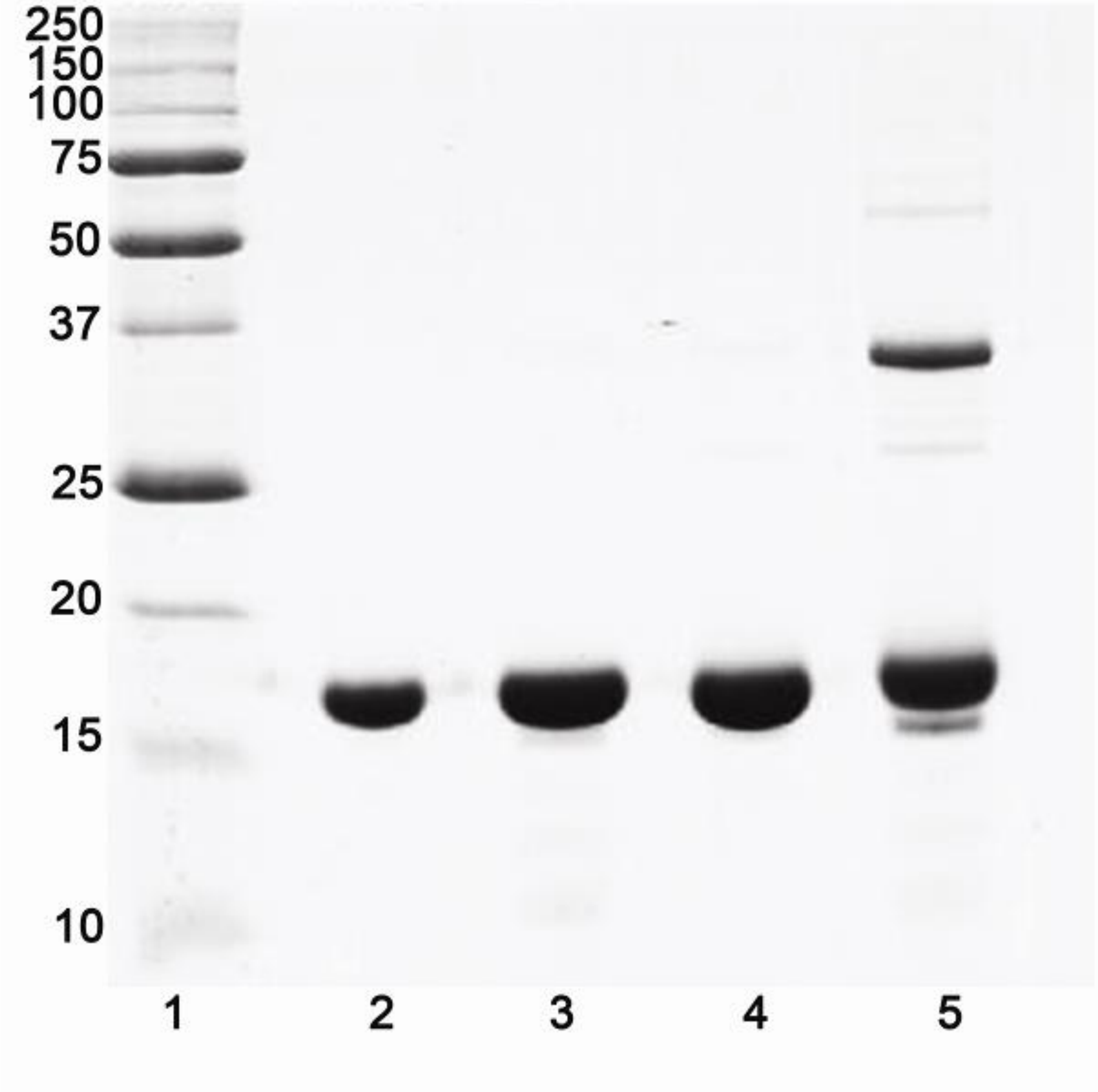

Figure S2: Crosslinking analysis of Bcl-xL. Bcl-xL was crosslinked using 1,6-Bis(maleimido) hexane (BMH) to confirm the presence of dimer species. The molecular markers (in kDa) are shown in lane 1, followed by apo form (lane 2), addition of 2 % OM (lane 3), 1mM BMH only (lane 4) and 2 % OM and 1mM BMH (lane 5). Lane 5 clearly indicates the presence of the dimer induced by OM and stabilized by BMH.

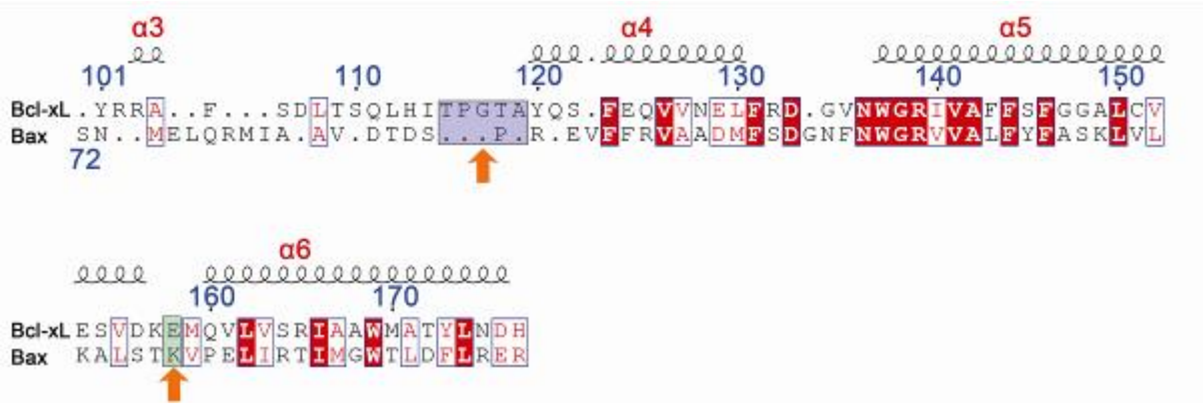

Figure S3: The structure-based sequence alignment of the α3 to α6 regions of Bcl-xL over Bax indicates differences in the α3-α4 loop region (shown within the blue box) while the other regions are similar. The residue Glu158/Lys128 in the α5-α6 loop corresponding to Bcl-xL/Bax which undergoes a major torsion angle change is shown within the green colored box. The residue numbers corresponding to Bcl-xL are shown above the sequences.

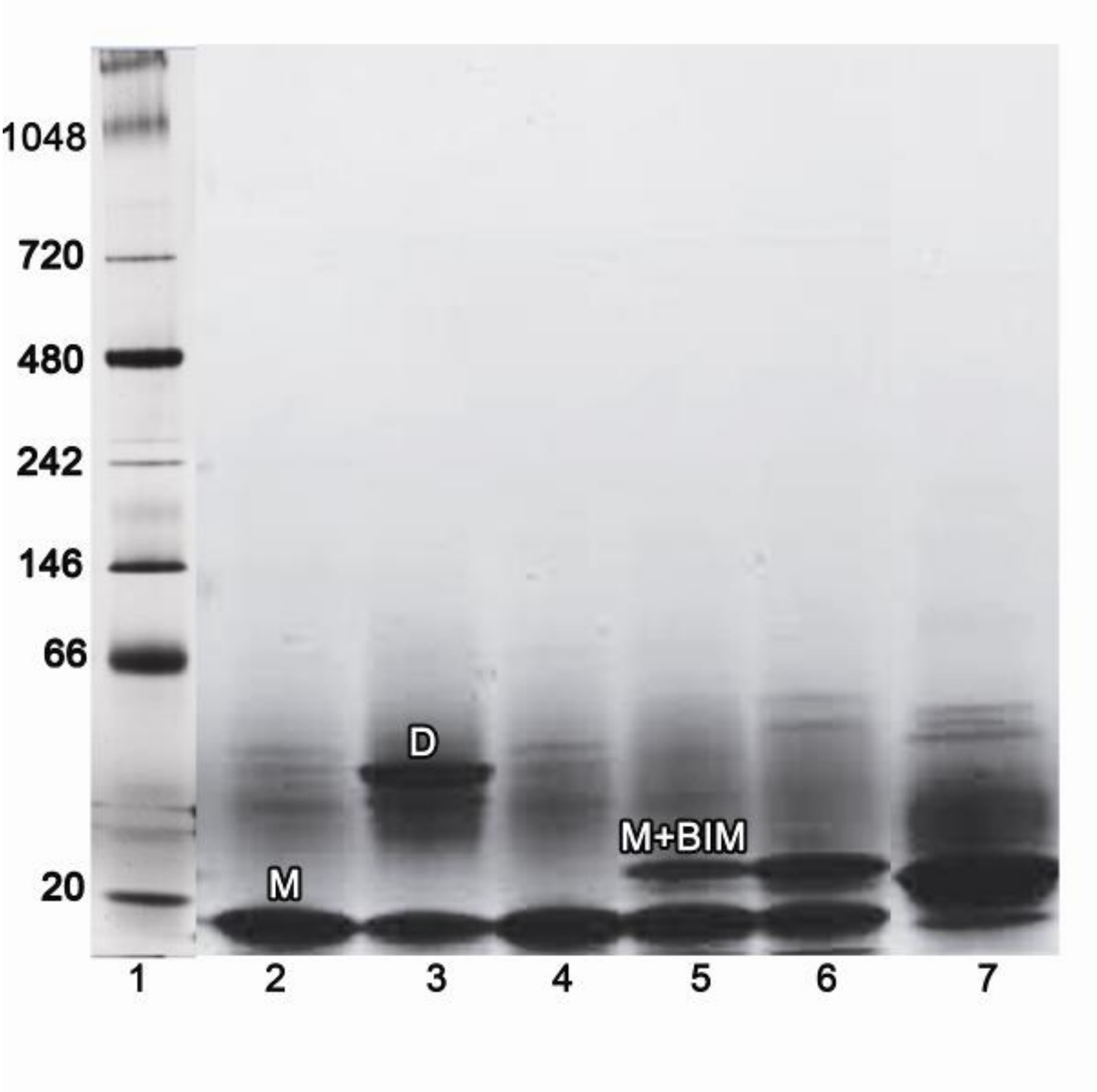

Figure S4: BN-PAGE illustrating the effect of CHAPS on Bcl-xL dimerization. The molecular weight marker (in kDa) (lane 1), followed by Bcl-xL (lane 2) ; Bcl-xL with 2 % OM (lane 3) to indicate the dimer position ; Bcl-xL with 1.5 % CHAPS (lane 4) ; Bcl-xL with 1.5 % CHAPS and BIM at molar ratios of 1:1, 1:5 and 1:10 are shown in lanes 5, 6 and 7, respectively. It could be seen that CHAPS does not induce Bcl-xL dimers, either alone (4) or in the presence of increasing concentration of BIM BH3 peptide (5, 6, 7). In comparison, the amount of monomeric Bcl-xL- BIM complex clearly increases with an increase of BIM concentration.

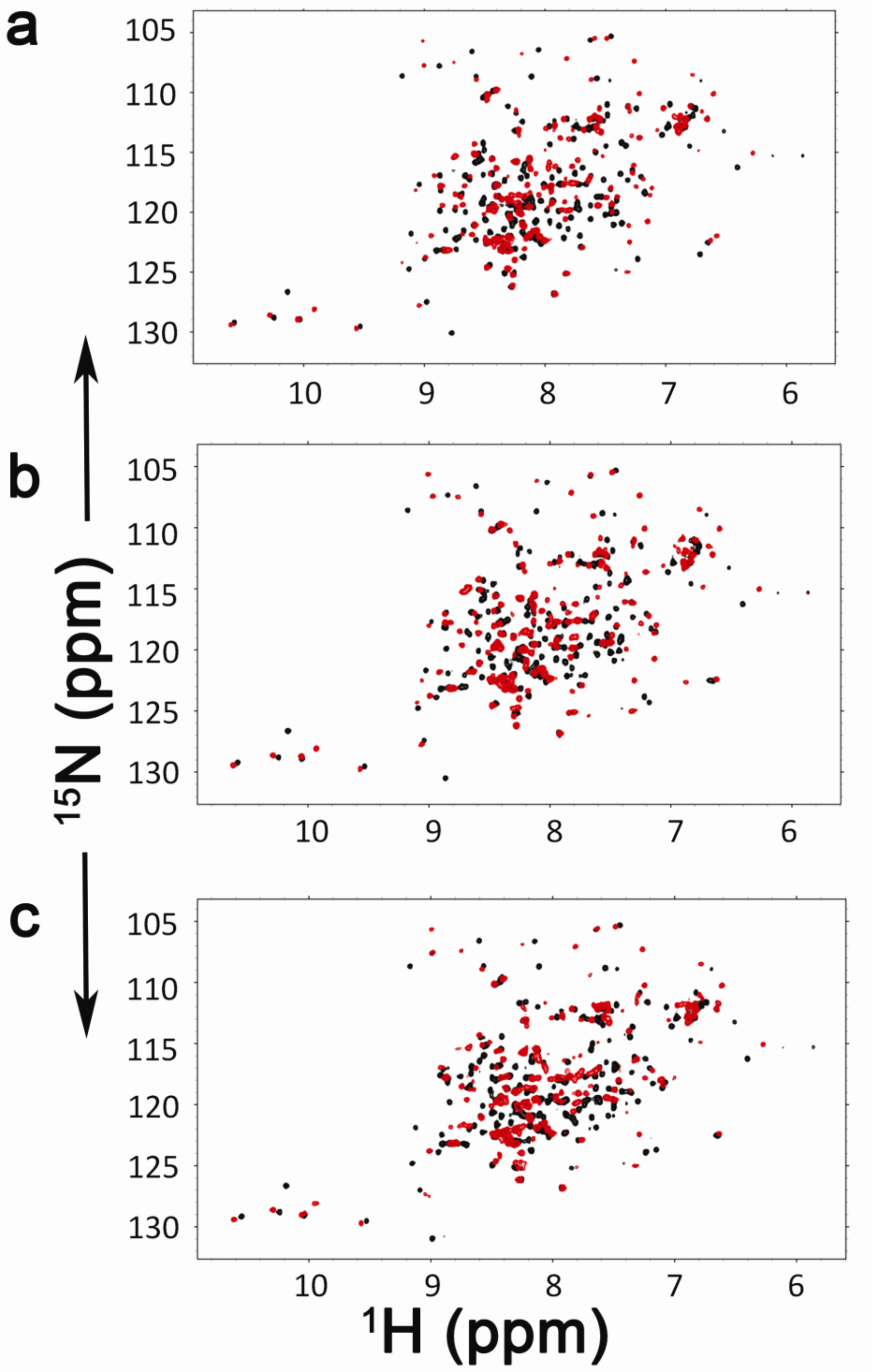

Figure S5: Overlay of 2D ^{1}H-^{15}N HSQC spectra of 0.2 mM ^{15}N-labeled Bcl-xL (a), Bcl-xL - E158P (b), and Bcl-xL - M159P (c) (black) saturated with BIM BH3 peptide (red) at a 1:2 molar ratio of protein: peptide. The NMR comparison reveals that the Bcl-xL mutant's E158P (b) and M159P (c) remain folded and bound to BIM BH3 peptide comparable with the WT form (a).

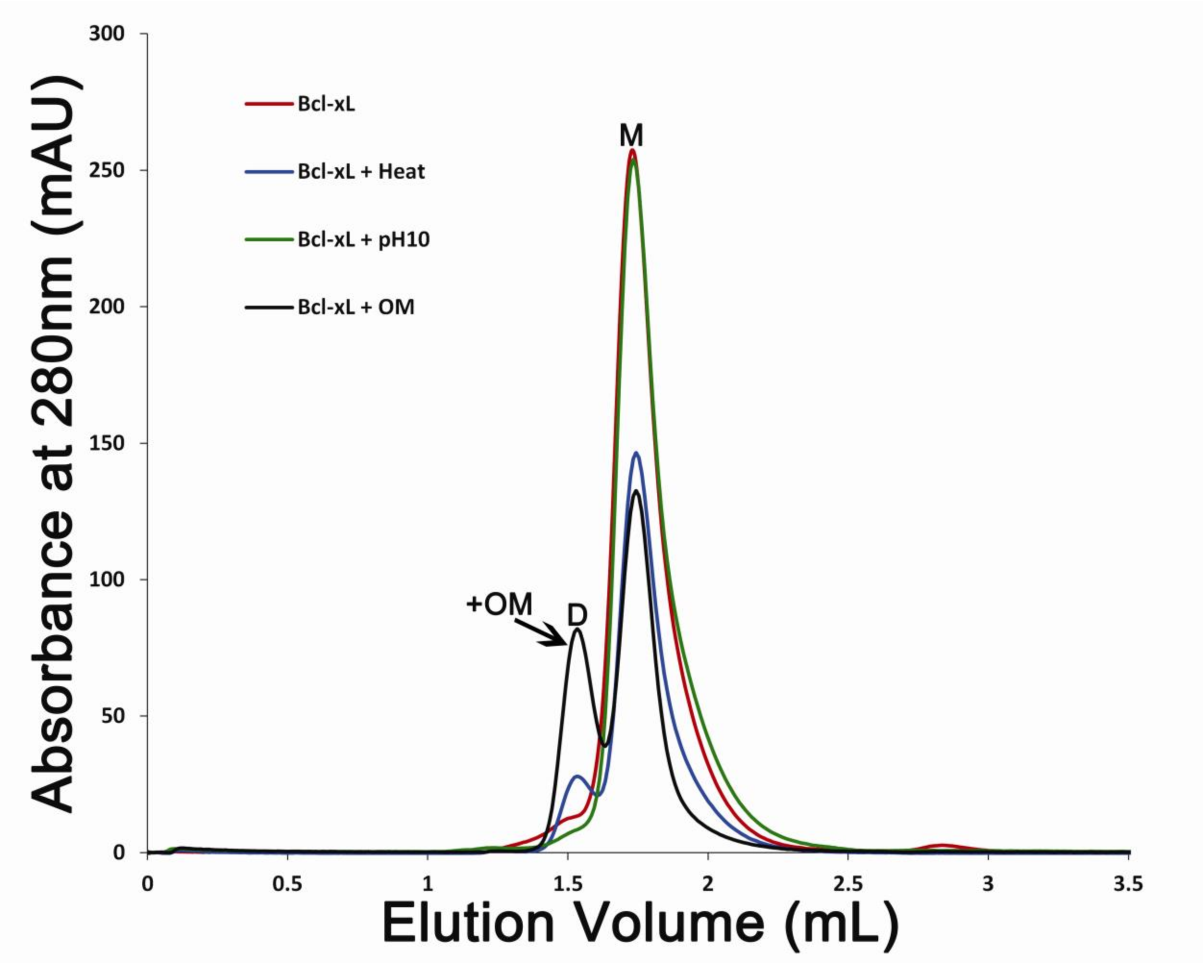

Figure S6: Effect of pH, heat and OM on Bcl-xL oligomerization**.** The Superdex 75 15/150GL (3 ml) analytical column profile of Bcl-xL treated with heat (55 ˚C) (blue line), alkaline pH (pH 10.0) (green line) and OM (2 %) (black line), indicating that the OM induced dimer is more prominent compared to others. The untreated Bcl-xL (red line) is also shown for comparison to indicate the position of the monomer.

Supplemental Tables

Table S1: The phi and psi torsion angles of the residue Glu158 of Bcl-xL in monomeric and dimeric forms. A similar tabulation of the equivalent residue Lys128 in the Bax structure is also shown.

Protein	Remark	Residue	Phi (in ˚)	Psi (in ˚)	PDB ID
Bcl-xL	Apo	E158	65.78	19.57	4EHR
Bcl-xL*	pH induced	E158	-91.69	-18.16	2B48
Bcl-xL*	OM induced	E158	-65.74	-34.46	4PPI
Bax #	Apo	K128	60.60	51.75	2K7W
Bax*	OM induced	K128	-70.61	-32.20	4BD7

* denotes 3DDS dimer; # solved by NMR, while others are solved by X-ray crystallography

Table S2: X-ray crystallographic data reduction and refinement statistics for 3DDS Bcl-xL induced by OM

**Data Reduction**	
Wavelength (Å)	1.0000
Space Group	I 4_1 3 2
Cell Dimensions	
a = *b* = *c* (Å)	153.630
α= β = γ (˚)	90
Resolution (Å)	44-2.85 (3.01-2.85) †
R_{merge}	0.086 (0.709)
Unique Reflections	7536 (1072)
Mean [(I)/σ (I)]	28.3 (5.3)
Completeness (%)	99.9 (100.0)
Multiplicity	38.8 (41.3)
**Refinement**	
Number of Reflections	7211
Resolution (Å)	27.0-2.85
R_{Work} / R_{Free}	0.2364 / 0.2687
No. of protein atoms	1099
No. of glycerol atoms	6
No. of water molecules	15
Average *B*-Factors ($Å^2$)	
Overall / Protein / Glycerol / Water	91.70 / 91.86 / 82.17 / 88.69
R.m.s deviations	
Bond lengths (Å)	0.005
Bond angles (˚)	0.982
**Ramachandran Statistics**	
Preferred Regions (%)	93.1
Allowed Regions (%)	5.4
Disallowed (%)	1.5

† Values in the parenthesis correspond to those in the highest resolution bin.
